# Flavors of the Earth: Bioprospecting and Potential of Agricultural Ingredients in Yogurt Production with a Focus on Sustainability, Quality, and Technological Innovation

**DOI:** 10.3390/foods14091497

**Published:** 2025-04-25

**Authors:** Carlos Eduardo de Faria Cardoso, Sofia Terra Silva, Maria Eduarda Flores Trindade, Monique de Barros E. Campos, Adriano Gomes Cruz, Francine Albernaz T. Fonseca Lobo, Anderson Junger Teodoro

**Affiliations:** 1 Postgraduate Program in Food and Nutrition (PPGAN), Federal University of the State of Rio de Janeiro (UNIRIO), Rio de Janeiro 22290-240, Brazil; sofiaterra@id.uff.br (S.T.S.); francine.lobo@unirio.br (F.A.T.F.L.); 2 Integrated Food and Nutrition Center (CIAN), Faculty of Nutrition, Fluminense Federal University (UFF), Niterói 24020-140, Brazil; mariaeduuardaflores@gmail.com (M.E.F.T.); moniquebarros.nutri@gmail.com (M.d.B.E.C.); 3 Postgraduate Program in Applied Health Products (PPGCAPS), Faculty of Pharmacy, Fluminense Federal University (UFF), Niterói 24020-140, Brazil; 4 Department of Food, Federal Institute of Education, Science and Technology of Rio de Janeiro (IFRJ), Rio de Janeiro 20270-021, Brazil; adriano.cruz@ifrj.edu.br

**Keywords:** bioprospecting, circular economy, functional food

## Abstract

There is a growing interest in promoting health and improving quality of life, which has led consumers to prefer foods that offer not only basic nutrition but also additional health benefits. In this space, yogurt has gained increasing attention due to its potential to deliver bioactive compounds and improve overall consumer well-being. As a fermented dairy product consumed globally, yogurt serves as an effective dietary base for nutritional enhancement through the incorporation of a wide range of primary agricultural products, including fruits, vegetables, cereals, and their respective by-products, including peels, seeds, and pomace. This review provides an overview of recent advances in yogurt biofortification using primary agricultural matrices and agro-industrial by-products within the framework of sustainable food systems and the circular economy. Significant increases in antioxidant activity and final phytochemical content are observed after the addition of ingredients to yogurt. Enrichment with dietary fiber from fruit peels or pomace also improved syneresis control and viscosity of the products. The microbiological viability of probiotic strains was maintained or increased in most formulations, and sensory acceptance remained favorable with enriched yogurts. These findings highlight the potential of agricultural matrices to enhance yogurt functionality, promoting sustainability and reducing food waste.

## 1. Introduction

In the face of the global environmental emergency, driven by the depletion of resources, the food industry is becoming increasingly aware of the importance of adopting sustainable practices with a view to reducing waste [1]. In the traditional food processing and production model, companies collect and extract resources, transforming them into final products that are eventually used by the end consumer, and their by-products are largely discarded. Currently, companies are becoming increasingly aware of the risks associated with this production model.

In recognition of this movement, there is a growing search for an industrial model that dissociates this “recipe” and establishes action plans to eliminate food waste using a model based on the concept of a circular economy in food production, but which also goes hand in hand with the Sustainable Development Goals (SDGs), implemented in 2015 by the United Nations (UN) 2030 Agenda [2].

The circular economy goes far beyond the recycling of materials, involving the adoption of different flows that allow rethinking the use of resources with a view to (FAO) promoting sustainable practices in the precision agriculture niche, where agri-food waste, co-products, and by-products become protagonists and are essential for the promotion of food diversity and security [3,4].

Agri-food products and by-products generated throughout the agricultural and horticultural food processing chain contain numerous nutritional and bioactive components that can be used in the incorporation and development of innovative food products, aiming to reduce waste, provide a more efficient flow, and improve the overall sustainability of food systems [5,6].

It is known that the food sector faces challenges related to consumer satisfaction, reflecting a growing trend that prioritizes nutritional, sensory, and health aspects of products that undergo some degree of industrialization [7]. The development of new food products increasingly focuses on health and practicality, leading these companies to seek innovations that meet these requirements efficiently, with a certain emerging “content” [8,9]. This pattern is especially noticeable in the dairy industry and its derivatives market. Because they have a versatile base both sensorially and technologically speaking, which allows for different forms of presentation, dairy products are widely used to incorporate by-products from different industrial segments, including horticulture, encouraging the inclusion of these components in formulations that are already conventionally accepted by the general population, such as yogurts and dairy drinks [10]. For products with greater nutritional value and physical–chemical quality required by law and the market, their enrichment is discussed through the incorporation of natural ingredients with improved functional and phytochemical properties in a movement known in the industry as “Bioprospecting” [11].

Bioprospecting involves the identification and exploitation of natural resources for commercial purposes [12], generating interest among manufacturers in incorporating their concepts into the development of new products that have market potential but also meet the demands of sustainability and consumer acceptability, as previously discussed, making it necessary to think of bioprospecting as a tool to find more sustainable solutions for food production and processing and to shorten the gap between these aspects [13,14,15].

The inclusion of direct products and plant by-products, for the formulation of yogurts, for example, is in line with the guidelines of the Food and Agriculture Organization of the United Nations (FAO), which encourage sustainable practices in the food industry to minimize waste and optimize the use of resources and inputs [16]. However, the integration of some primary and by-products of plant origin into yogurts still faces technical and sensory challenges that need to be overcome to ensure consumer acceptance and the commercial viability of the formulated product, requiring the promotion and improvement of production technologies.

In this scenario, this article describes the current panorama of the processing and production of bioenriched yogurts, providing timely updates through bibliographic research and critical analysis with a qualitative and exploratory approach [17,18] on how new products are being formulated, from the emerging perspective of the circular economy, bioprospecting, and the waste reduction of direct/primary products (pulps, juices, extracts and flours) and by-products (peels, seeds, pulps, press cakes) from horticultural production (fruits, vegetables, legumes, spices, etc.), aiming to reduce their environmental and economic impact. It is noteworthy that these products can be incorporated as ingredients in foods with functional appeal or with nutraceutical potential. These solutions align with the concept of a circular bioeconomy, allowing for the reduction of environmental, social, and economic costs, increasing economic competitiveness, and alleviating poverty and hunger [19].

## 2. Methods

This review may provide some new insights into the nutritional, functional, sensory, and technological aspects of new dairy products formulated under the emerging perspective of a circular economy, bioprospecting, and waste reduction of products from horticultural production. This may lead to new explorations of the relationship between the addition of these “actors” and the quality of the final product. The methodological planning involved the following steps: (a) definition of the research problem; (b) careful selection of databases to search for relevant studies published in the last decade; (c) establishment of inclusion and exclusion criteria; and (d) critical analysis and discussion of the findings. Although this is not a systematic review, the search for greater transparency and traceability in data collection was supported by principles adapted from the PRISMA (Preferred Reporting Items for Systematic Reviews and Meta-Analyses) methodology, especially with regard to the stages of identification, screening, eligibility, and inclusion of studies [20].

The search was carried out in the following databases: Google Scholar©, SciELO©, ScienceDirect©, PubMed©, Scopus©, and Web of Science©, covering publications between 2013 and 2025. The following descriptors and combinations were used: “dairy products”, “yogurt”, associated with terms such as “lactic fermentation”, “consumer market”, “bioenrichment”, “fortification”, “nutritional composition”, “chemical composition”, “bioactive compounds”, “antioxidant capacity”, “technological properties”, “functional properties” and “biological properties”. The following were considered for inclusion: original articles and reviews, published in Portuguese, English, or Spanish, available in full (with free or paid access). The following were excluded: abstracts, works published in event proceedings, course completion papers (TCC), dissertations, theses, and articles that were not in the selected languages.

## 3. Food Production, Waste, and Sustainability: Bases for the Valorization of Ingredients Obtained from Agricultural Processing

By 2050, the demand for food matrices is expected to increase significantly, by approximately 55%, due to factors related to population growth, the economic development of developed and underdeveloped countries, and urbanization [2,21]. However, this massive expansion will have negative effects on a wide range of sectors, such as biodiversity, the economy, and even health systems. In this context, it is imperative that the increase in food supply be accompanied by public policies and sustainable production strategies, where such approaches must incorporate principles of the circular economy, favoring practices that reduce environmental impact and, at the same time, meet the technological and consumption demands of contemporary society [21]. In order to minimize consistent losses related mainly to inadequate management, poor drainage, and food waste, it is widely considered to adopt strategies that aim to reduce production costs but at the same time contribute to increasing the efficiency and optimization of food systems, strengthening biodiversity, and improving aspects related to food security [21,22,23].

Food waste is a global issue. Estimates suggest that more than US$ 1 trillion in food is wasted each year, which represents more than one-third of all food produced globally, using more than a quarter (28%) of the world’s agricultural area. In the context of biodiversity, food waste is also responsible for about 8–10% of greenhouse gas emissions [21,23]. As environmental impacts accumulate throughout the life cycle of food products, food waste at the consumer level represents the greatest burden on the survival of civilization. It sheds light on the magnitude of food waste and the prevalence of household food waste across all continents, regardless of country income levels [23]. This growing attention to food loss and waste is reflected in the SDGs, which are defined in the UN 2030 Agenda [2]. Specifically, goal #12 aims to halve global food waste per capita at retail and consumer levels and reduce food losses along production and supply chains (including post-harvest losses at all stages of production/consumption) by 2030. Reducing food loss and waste can also contribute to achieving other SDGs, such as #2 “Zero Hunger”, SDG #6 “Sustainable Water Management”, SDG #13 “Climate Change”, and SDG #15 “Terrestrial Ecosystems, Forests, Biodiversity” [2]. Recognizing this universal problem, the industry has been joining forces with the scientific community, focusing increasingly on the optimization and use of agro-industrial resources, such as direct products and by-products. There is a growing interest in research on the formulation of new products incorporating elements derived from agricultural practices and their potential benefits.

## 4. Technological Advances and Market Projections in the Dairy Industry for New Products at the Interface of Bioprospecting and the Circular Economy

In the broader context of the management of agro-industrial products and by-products, technological advances stand out, creating an interface in the axes of biodiversity and circular economy, which fosters the practice of Industry 4.0 in the development of new food products in the industrial framework. Industry 4.0 is transforming the production sector in general, and the food sector is no different [24]. Through the integration of intelligent, interconnected, and independent systems that revolutionize traditional production practices and drive the adoption of innovations that address the sustainability and healthiness of products, as required in the current market scenario [25], technologies such as artificial intelligence and robotics improve supply chain efficiency, reduce waste and risks, and increase traceability and food safety, which are essential aspects of guaranteeing product quality and minimizing environmental impacts [26,27].

In this scenario, bioprospecting emerges as a promising strategy for the discovery of new bioactive compounds from natural sources, including primary products and agro-industrial by-products derived from vegetables and fruits. The search for bioactive compounds with functional properties, such as flavonoids, polyphenols, prebiotic fibers, and other components, allows the development of healthier and more nutritious formulations while contributing to the sustainability of the production chain. In addition to enriching the nutritional composition of foods, bioprospecting allows the valorization of these products by transforming them into ingredients, reducing waste, and encouraging practices that enhance the circular economy [28,29].

The incorporation of these ingredients rich in bioactive compounds into food matrices not only improves the properties (technological and nutritional) of the products but also provides health benefits to consumers and new sensory experiences [29]. This advancement has driven innovations in food formulation, which seek to differentiate themselves in the market by offering improved technological, sensory, and bioactive properties and experiences [30,31]. From this perspective, the dairy industry exemplifies this evolution well by developing bioenriched yogurts and dairy drinks [11,32].

These advances demonstrate that the contradiction between science, technology, and innovation has been a fundamental pillar for the development of healthy and sustainable products, mobilizing a rearrangement of industries in this movement. By supporting productive efficiency, innovation in obtaining new ingredients, and the implementation of strategies to reduce environmental impacts, the food industry is moving towards a more sustainable model in which technology and bioprospecting become essential and complementary allies in promoting health and creating more natural and beneficial products for the consumer [33,34]. It is worth noting that a sedentary lifestyle, poor eating habits, and the profile of modern diseases are worrying factors for the current generation, as it is already known that these behaviors determine their quality of life, in general, as well as their longevity [35,36,37], which generates concerns about maintaining health; combined with advances in health studies, this population is led to seek healthier foods, encouraging the redesign of the most diverse market segments [38].

The size of the dairy market is estimated at US$ 620 billion in 2024 and is expected to reach US$ 768.80 billion by 2029, with a growth of 4.40% during the forecast period 2024–2029 [39]. The growth of the segment is attributed to the consumption of food prepared outside the home, with greater nutritional and sensory appeal [38,40].

Among the most popular and culturally relevant functional products are fermented dairy products, especially yogurt, which combine tradition, nutritional value, and unique technological properties [41]. Yogurt is produced by fermenting natural milk (from cows, buffaloes, and goats, for example) with specific bacterial cultures, such as *Streptococcus thermophilus* and *Lactobacillus delbrueckii* subsp. *bulgaricus*, which metabolize lactose (milk sugar) and generate lactic acid, which is responsible for the sensory characteristics of the product [14,42]. This technological evolution and innovation perceived in the dairy industry has allowed the development of improved yogurt formulations, adjusting the composition of the ingredients to improve nutritional, technological, and sensory properties. Furthermore, bioprospecting has been a fundamental strategy in the reformulation of already commercialized products, allowing the incorporation of functional components derived from the horticultural industry and promoting the sustainability and use of these matrices [11,32].

The production of bioenriched yogurts illustrates how the convergence of science, technology, and innovation can create products that meet market and sustainability demands. The new production standard, through the advancement of technologies brought by Industry 4.0, favors the production of improved yogurts since their production involves the rigorous control of factors such as the quality of the base raw material, choice of starter cultures, fermentation equipment, temperature, and processing time, ensuring probiotic specifications and the standardization of the sensory characteristics of the final product [14,15]. The yogurt market has also diversified to meet different consumer profiles, including options such as traditional, Greek, shaken, drinkable, and frozen yogurt, each with variations in texture, acidity, protein content, and biological benefits and specific phytochemical enhancement [15]. This segmentation reinforces the importance of research and innovation to develop products aligned with consumer demands, maintaining a balance between flavor and functionality without sacrificing sustainability in their processing.

In this way, the combination of bioprospecting, technology, and the innovative formulation of new products for the food market allows production sectors, especially the dairy sector, to increase their competitiveness, especially in the segment of foods with functional appeal, by adding nutritional value and promoting conscious and sustainable consumption. By integrating nutritional enrichment strategies and technological control, the yogurt industry exemplifies how innovation can contribute to the creation of products that meet the needs of consumers and the environment.

## 5. Trends and Effects of Enrichment with Vegetables Designated as Ingredients in the Development of Functional Yogurts

Bioactive substances, despite their low bioavailability, are mainly derived from the chemical arrangement of the molecule and the processing methods applied to the matrix, and they play important roles in several physiological processes and offer health benefits to the consumer, justifying their incorporation, under the name of ingredient, in different base foods [43,44,45,46]. In general, this addition involves increasing the levels of nutrients and bioactive substances to acceptable levels so that they can exert a biological effect, potentially acting in the process of promoting health and reducing the occurrence of chronic diseases, such as cardiovascular diseases, cancer, diabetes, and neurodegenerative diseases [45,46,47,48,49].

The enrichment of foods already recognized by consumers in the market with ingredients derived from primary products and agro-industrial by-products rich in these substances not only improves the health and well-being of the consumer but also aligns with the profitability and sustainability goals that have been incorporated into the current industrial production model [16,50].

Natural yogurts are widely recognized for their numerous health benefits, especially with regard to human nutrition and well-being [15]. Among these benefits, the presence of substances such as peptides, proteins, and some important short-chain fatty acids formed in the fermentation process that are essential for strengthening the immune system, for example, stands out [46,51].

**Figure 1:**
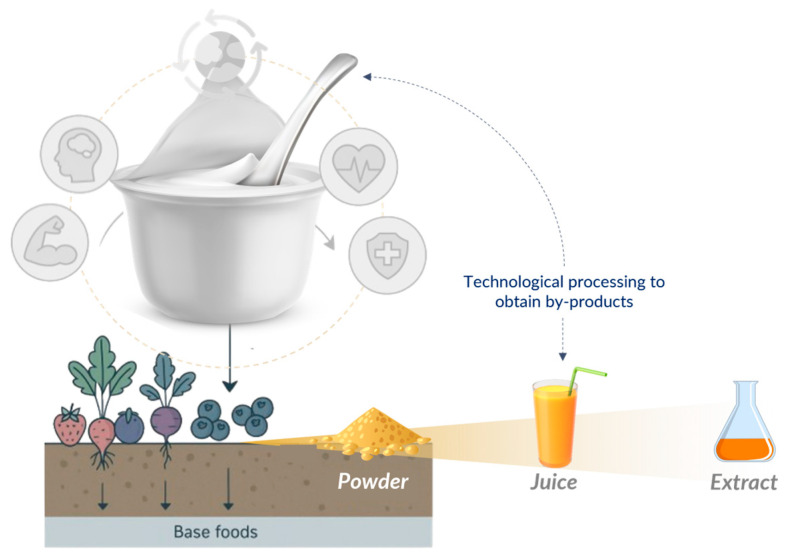
Agro-industrial primary products and by-products as a source of functional ingredients for yogurts. Source: Authors, 2025.

However, it has been found that the supply of bioactive compounds, such as phenolics, flavonoids, anthocyanins, etc., imposes certain limitations on the functional properties of natural yogurt [52,53,54], which makes this product a target for industrial desires to incorporate elements and add value. In this context, the enrichment of yogurts with natural functional ingredients has gained prominence in the scientific community [15], where the addition of these compounds to dairy products can occur through the direct inclusion of ingredients rich in these substances, such as jellies, purees, pulps, flours, or powders obtained from primary products and by-products of the processing of fruits, vegetables, and spices. From a commercial point of view, yogurt stands out as one of the most versatile foods, presenting high compatibility with various ingredients. This characteristic not only increases its acceptance by the consumer but also opens space to enhance its nutritional benefits, favor biological activity, and improve its technological and commercial standards (Figure 1).

### 5.1. Applications in Yogurt: Qualitative Analysis of the Evidence

The valorization of agroindustrial elements (primary products and by-products) has stood out as a sustainable strategy to reduce waste and add nutritional value to food by designating these components as “ingredients” in the food industry. Currently, the addition of synthetic additives in the preparation of products, such as yogurt, aims to correct technological flaws or improve the characteristics of the final product, including in the formulations, by using substances that have stabilizing, thickening, emulsifying, and coloring action, among others. However, the addition of these synthetic ingredients/additives can have harmful effects on human health [55], leading consumers to seek more natural options that meet the notable demand for foods that have a transversal scope with sustainability and healthiness. In the context of synthetic additives, their use requires limitations regarding their minimum and maximum use, resulting in changes in the market and in the products offered, which is mainly represented by the “clean label” movement [56]. This current trend has stimulated scientific research aimed at finding alternative ingredients to synthetic additives.

In the conventional industry, the additives incorporated in the manufacture of yogurts are configured as a subset of edible raw materials for the processing, manufacturing, treatment, and preparation of a product designated for sale, such as yogurt [57]. Therefore, several studies have focused their efforts on demonstrating, through evidence, that there are several possibilities for improving the physical, chemical, sensory, and nutritional characteristics of yogurts, especially with the use of different matrices of plant origin, in the form of primary product or by-product.

In order to understand the current panorama of applications of new ingredients derived from horticultural processing in yogurts, a summary of the main studies published between 2013 and 2025 was prepared. Table 1 presents a detailed survey of research that incorporated primary materials and by-products from agricultural practices such as pulps, purees, extracts, flours, peels, seeds, residual pulps, and pressing cakes, for example, in yogurt formulations, analyzing their effects on physical–chemical, microbiological, sensory, and functional parameters.

It is observed that the improvement of the traditional formulation of yogurts is a constantly growing market, which requires the prior development of new ingredients with the aim of enhancing the bioactive effects of these foods. These bioactive components can be used in the development of innovative functional products, a theme addressed by most of the studies, produced in the last decade highlight the natural alternatives capable of meeting the growing demand for functional ingredients added to these foods. Currently, the world is turning to the exploration of organic agricultural products (whether primary or secondary), which constitute a great burden of environmental potential, for inclusion in the food industry, directly impacting the quality of the final product [88].

However, the application of processing technologies to these potential ingredients plays a fundamental role in improving the final product, especially on the yogurt production scale. This is because, depending on this initial stage of production, a favorable or unfavorable result can be achieved in the formulation, since it is known that various processing methods applied to both the added ingredients and the yogurt itself directly influence the stability and bioavailability of phytochemical compounds throughout the production chain.

Drying, for example, often used in the production of fruit and vegetable powders, can cause significant losses of heat-sensitive compounds, such as vitamin C and anthocyanins, especially when carried out at high temperatures [89]; this directly impacts sensory elements, such as color, and functional elements, such as the antioxidant capacity observed in the final formulation. On the other hand, freeze-drying can be used as a technology that more effectively preserves phytochemicals due to the absence of intense heat in the methodological dynamics, with the same being observed for products obtained by drying in a foam mat, for example [90]. In this same line of reasoning, related to the use of temperature, the thermal treatment of yogurt, such as the pasteurization of the milk used as a base, can partially degrade polyphenols and carotenoids present in ingredients added later through the use of heat [91]; however, this step is configured as an essential procedural element to stimulate the beginning of the growth of the lactic culture by reducing the oxygen content of the milk, in addition to directly influencing the increase in the viscosity of the yogurt and obtaining a good texture of the final product due to the management of intrinsic factors, such as protein rearrangement, formation of the lipid emulsion system, etc.

In terms of methodology/process, other technologies, such as ultrasound [73,92] and encapsulation [87], have been explored both for the extraction and preservation of phytochemicals in plant ingredients that can be incorporated into yogurt with functional appeal [92].

Furthermore, fermentation, a process inherent to yogurt production, can promote the biotransformation of phytochemicals, enhancing their antioxidant activity or altering their chemical form, which directly impacts their functionality. This interaction between probiotic microorganisms and added compounds can be beneficial, as observed in formulations with pomegranate and grape seed powder, for example [67].

Therefore, the choice of processing method should consider not only the stability of the phytochemical compounds present in the added ingredients but also their interaction with the dairy matrix and the microorganisms present in the yogurt, aiming to guarantee the functionality and nutritional quality of the final product. In this context, it is important to consider that the addition of functional ingredients to yogurt, in addition to impacting nutritional and functional aspects, can also directly influence the physicochemical parameters of the product since these changes occur due to the intrinsic characteristics of the added compounds, as well as their interactions with the fermenting microbiota and the dairy matrix. Therefore, understanding the effects of enrichment on these parameters is essential to ensure product stability, sensory acceptability, and technological performance during storage and consumption.

### 5.2. Effects on pH and Titratable Acidity

pH and titratable acidity are essential parameters in the analysis of acidic foods, indicating quality and aligning with microbiological and sensory standards [93]. It is known that the fermentation of milk by lactic bacteria results in the production of lactic acid, reducing the pH and giving yogurt its characteristic texture and flavor. The addition of natural ingredients can modify these parameters, impacting the desired balance for the product, which is influenced by the type, concentration, and duration of storage [94]. Taking Brazilian legislation as an example, a pH limit of around 4.5 and a titratable acidity between 0.6–1.5 g% of lactic acid per 100 g/mL of the product are recommended to ensure sensory acceptance and stability [94]. After fermentation, lactic bacteria continue to metabolize during storage, influencing these values [61,63]. The inclusion of citrus and tropical fruit pulps, such as passion fruit [82], orange [60], and strawberry [83], often leads to a decrease in pH and an increase in titratable acidity since these ingredients are naturally rich in organic acids, such as citric, ascorbic and malic; the same movement was observed with the addition of apricot fiber powder [70] and chokeberry fractions (puree and juice from the pulp and pomace powder) [84]. This effect can accelerate casein coagulation, making the yogurt thicker and more stable. On the other hand, dietary fibers extracted from fruits and vegetables can have a buffering effect on the acidity of the medium, resulting in a yogurt with a higher pH and a less acidic sensory flavor. This mechanism occurs as a spring-loaded anchoring system for the casein micellar network, increasing the water-binding capacity and improving the globular microstructure of the yogurt by the formation of smaller pores [15,95,96,97]. This effect was observed with the addition of apple pomace fibers and grape skins, which appear to interfere with the availability of substrates such as lactose for fermentation, modulating the production of lactic acid by lactic bacteria [59,63].

By creating a parallel between the presence of phenolic compounds and the presence of bacterial species, it is possible to relate the interaction of these factors and how the presence of these compounds can influence the activity of fermentative bacteria, thus impacting the acidification rate of yogurt [80,82,83,85], for example. Extracts obtained from spices and herbs [75,77] can partially inhibit the activity of lactic bacteria due to their antimicrobial properties, resulting in yogurt with lower acidity. However, when these compounds are associated with sugars and flavonoids, they can act as prebiotics, stimulating the growth of probiotic cultures and increasing the production of organic acids, which modifies the technological, nutritional, and sensory properties of the final product.

### 5.3. Microbiological Viability

The microbiota of yogurt plays an essential role in both its sensory and technological quality, as well as its functional value. The maintenance of the viability of lactic acid bacteria, such as *Lactobacillus delbrueckii* subsp. *bulgaricus* and *Streptococcus thermophilus*, can be positively or negatively impacted by enrichment with horticultural ingredients, depending on the chemical composition. Regulatory standards in countries such as the USA, Europe, and Brazil stipulate that yogurt should contain live cultures greater than 10^6^ CFU/mL for health benefits [94,98,99].

The increase in the count of probiotic microorganisms was observed with the addition of pulps and extracts rich in fiber and phenolic compounds that act as substrates for bacterial growth. For example, the inclusion of pomegranate and beetroot extracts [66] and pineapple peel extracts [58] resulted in the increased viability of *Lactobacillus acidophilus* and *Bifidobacterium* spp., suggesting a prebiotic effect of these ingredients. This can be explained by the fact that some insoluble fibers and oligosaccharides present in fruits and vegetables can be selectively fermented by these beneficial bacteria, stimulating their proliferation [85]. The same was observed for yogurts enriched with pomegranate seed powder and purple grape, with the logarithmic cfu/g count of *L. delbrueckii* subsp. *bulgaricus* increasing at the end of the fermentation period of the product [67]. However, some bioactive compounds, such as tannins and flavonoids present in plant extracts, may have antimicrobial properties, reducing the viability of lactic cultures, as observed in a product formulated with the addition of passion fruit juice [82], elderberry extract, and the powdered pulp of lentinus edodes [72]. This effect was also observed in yogurts enriched with green tea and black tea Muniandy, Shori, and Baba [75], which showed a slight decrease in the Lactobacillus count, possibly due to the interaction of polyphenols with bacterial cell membranes. In addition, the presence of organic acids in tropical fruits may inhibit the growth of contaminating microorganisms, contributing to the preservation of the product without the need for artificial additives.

### 5.4. Technological Properties

The microstructure of yogurt, characterized by a complex network of casein micellar aggregates, shapes its texture and consistency, influencing sensory attributes and consumer preference [100]. Important physical variables such as texture, viscosity, water retention, and syneresis are critical in determining yogurt quality and consumer acceptance [15]. Syneresis, the gradual expulsion of water during storage, is exacerbated in yogurts enriched with liquid extracts, such as those observed in pomegranate peel and passion fruit juice, due to decreased matrix viscosity and structural destabilization [74,82]. On the other hand, enrichment with walnut shell extract increases stability by reducing syneresis [78], with the same effect observed with the addition of insoluble fibers from fruit peels, such as orange [60] and apple [63], where there is an increase in the viscosity of the yogurt, making it thicker and creamier. This occurs because these fibers act as stabilizing agents, reducing syneresis (whey release) and improving water retention capacity.

In addition, the structure of the yogurt gel can be affected by the pH and acidity generated by the addition of vegetable ingredients. Yogurts enriched with acidic fruits tend to be firmer, as acidity favors the denaturation of milk proteins and the formation of a more cohesive protein network due to a more stable environment. On the other hand, ingredients rich in lipids and phenolic compounds can interfere with protein coagulation, resulting in less firm yogurts.

In theory, improving yogurt texture involves increasing the total solids content to enhance viscoelastic characteristics and water retention, thereby reducing syneresis [101]. Enrichment with grape pomace, orange fiber, apple pomace, and white plum powder significantly reduces this movement, attributed to their hygroscopic properties [53,59,60,61,62,63,64,65]. Pectin-rich orange fiber influences the gel properties of yogurt, with lower fiber concentrations reducing syneresis and fine fibers promoting stabilization of the casein network [59]. These improvements in microstructure and rheological properties further increase the binding capacity of water-insoluble fiber fractions in yogurt formulations.

### 5.5. Phytochemical Enhancement

Enriching yogurts with natural functional ingredients significantly increases their phytochemical composition and antioxidant capacity, thereby enhancing their nutritional value and health benefits. While conventional yogurt already offers health benefits such as antioxidant properties and immune system support through its fermentation process and natural components, the addition of bioactive compounds such as phenols, flavonoids, and anthocyanins further enhances these benefits [46].

Incorporating extracts and infusions from various sources into yogurt formulations has been shown to increase the concentration of total phenolic compounds, flavonoids, and other phytonutrients. For example, additions such as pomegranate peel extract, grape pomace juice and powder, passion fruit juice, herbal extracts, and mango and banana powders have been shown to significantly increase the antioxidant potential of yogurts [59,74,75]. These ingredients interact directly with reactive oxygen species (ROS), thereby increasing the antioxidant capacity of the product, as evidenced by assays such as ABTS, DPPH, and FRAP [61,62,74,75]. Furthermore, innovations in processing techniques, such as the freeze-drying of grape pomace, have been shown to effectively preserve bioactive components, leading to higher levels of phenolic compounds and increased antioxidant activity in yogurts [61]. These advances not only improve the nutritional profile of yogurts but also increase their sensory appeal and consumer acceptance due to the perceived health benefits associated with antioxidant-rich formulations. In theory, incorporating natural extracts and by-products into yogurt represents a promising strategy to develop functional foods with enhanced bioactive properties, meeting both consumer demand for health-promoting products and industry goals for sustainable innovation in food processing. It is worth noting that the use of these by-products is not without challenges, since it is essential to ensure the stability of the antioxidant compounds of interest over time in the formulation of enriched yogurts. Factors such as exposure to oxygen, pH variation, storage temperature, and interactions with milk components can affect the retention and activity of the added phytochemicals.

### 5.6. Sensory Impact

The sensory appeal of yogurt is closely linked to its flavor compounds, which are derived from the lipolysis of milk fat and the microbial transformations of lactose and citrate during the fermentation process. Admittedly, there is a range of more than 100 volatile compounds that act systematically, contributing to the characteristic aroma and flavor of the product, with lactic acid, acetaldehyde, diacetyl, and acetoin among the main contributors [102]. The complexity involved in the “flavor” behind the product continues to shape innovations in production, ensuring that products remain tasty and nutritionally robust and accepted [102,103]. The sensory impact of adding products from the processing of agricultural matrices in the form of ingredients is one of the most relevant factors in the consumer’s acceptance of a product. Modifying flavor, aroma, color, and texture can influence consumer preference, making the product more or less attractive. In the dairy segment, consumers prefer homogeneous yogurts with a viscous body and without syneresis, which makes them easier to consume with a spoon, for example [48].

Enrichment with ingredients in the form of powders, flours, extracts, purees, etc. can improve the flavor and aroma of the product, giving it specific notes with a fruity and fresh tone and increasing the complexity of the sensory profile. Color is a crucial sensory attribute that impacts the acceptance of foods. In dairy products such as yogurt, natural pigments extracted from botanical sources are increasingly used as food colors, providing nutritional and sensory benefits. For example, the addition of beetroot extract with lemon improves the colorimetric properties of yogurt, changing its hue to reddish tones and enhancing the desired color profile, potentially increasing consumer appeal [76]. The same effect was observed for yogurts enriched with sweet potato powder [66]; black carrot powder [73]; and chokeberry juice, puree, and pomace powder [84].

Flavor and texture are also critical sensory dimensions in yogurt. Studies have shown that the enrichment of yogurts with fruit and vegetable extracts, powders, pulps, and juices significantly influences these attributes. Yogurts enriched with ingredients such as white cranberry powder, pomegranate and rosewood seed powder, purple grape juice, and passion fruit juice generally receive positive sensory scores compared to plain yogurt controls [59,62,65]. However, the sensory results of enrichments may vary. Some additives, such as walnut and almond extract or orange fiber, may not achieve desirable sensory profiles in yogurt formulations [60,78]. In general, the addition of ingredients such as tropical fruit pulps (passion fruit, strawberry, acerola) is well accepted due to the natural sweetness and freshness they add to the final product. On the other hand, the addition of plant extracts with more bitter or astringent flavor notes, such as green tea and pomegranate peel, may reduce sensory acceptance. This highlights the importance of carefully selecting ingredients and optimizing formulations to maintain or enhance sensory qualities while providing functional benefits. This perception was observed by Lee et al. [104] who, when sensorially evaluating a yogurt supplemented with hydroponic ginseng root extract, found that acceptability as a yogurt product was negatively affected at a concentration above 1.0%, corroborating that depending on the nature of the added product, the final “flavor” of the product may be impacted, establishing a contradiction with the benefits associated with it in terms of nutrition. Developing successful enriched yogurts requires balancing these factors to meet nutritional goals and exceed sensory expectations, thus improving their market appeal and competitiveness.

## 6. Limitations and Challenges of Bioprospecting in the Dairy Segment

The enrichment of yogurts with vegetable ingredients promotes a series of modifications in the physicochemical, microbiological, and sensory properties of the product, as we have seen previously. These changes can be advantageous when well balanced, but they require strict control to ensure the stability and acceptance of the yogurt by the consumer. However, there is some skepticism regarding the credibility of functional food claims. The regulation of functional and enriched foods varies from country to country and can directly impact the commercial viability of bioenriched products [105]. In Brazil, for example, the National Health Surveillance Agency (ANVISA) establishes that any product with functional claims must have scientific proof and meet minimum standards of composition and food safety [106,107]. In the United States, the Food and Drug Administration (FDA) adopts similar guidelines, requiring that health benefits be proven by clinical studies and that the product be properly labeled to avoid fraudulent claims to the consumer. In the European Union, the European Food Safety Authority (EFSA) imposes strict requirements for the approval of new ingredients with functional claims, which may make it difficult for products with this more “innovative” profile to enter the market [106].

In addition to regulatory challenges, the commercial acceptance of enriched yogurts depends on the consumer’s willingness to pay for these products, since consumers are increasingly inclined to choose foods that offer health benefits, even if they cost a little more [38]. However, the price needs to be carefully balanced so that the additional cost of the formulation does not drive away potential buyers.

The bioenrichment of yogurts with vegetable by-products represents an innovative solution to promote the improvement of emerging niches presented by the world population, anchored in sustainability and added nutritional value with a focus on healthiness. However, to ensure consumer acceptance and the commercial viability of these products, it is essential that the industry invests in research and development to optimize formulations, ensure sensory stability, and comply with international regulations. Furthermore, marketing strategies that emphasize the health benefits and sustainability of these products can strengthen their market position, making them a preferred choice for consumers concerned about healthy eating and environmental impact. In this way, yogurts enriched with plant-based by-products can play a key role in building a more sustainable and innovative food system.

## 7. Conclusions, Future Projections, and Highlights

Technological prospecting is increasingly vital to guide strategic decisions in the food sector, especially for organizations and niche industries that want to navigate a competitive technological landscape. By actively engaging in technology exploration, companies can anticipate future trends and prepare to adapt proactively. This vision allows them to maintain a competitive advantage, optimizing time and resources in a constantly evolving market environment.

Looking to the future, the dairy market, particularly the yogurt segment, is experiencing continued growth. Advanced technology allows consumers to easily access detailed information about products, promoting greater awareness about the origins of products and the sustainability practices involved in their production, which is shaping market expansion. In short, leveraging technological advances for strategic planning and meeting consumer demands in terms of transparency and sustainability will be crucial to sustaining growth and competitiveness in the dairy industry.

## Figures and Tables

**Table 1:** Effects of including vegetable ingredients and by-products in yogurts: qualitative review of research published between 2013 and 2025.

			Main Effects Observed				
Ref.	Ingredient	Added Fraction	Functional/Nutritional	Phytochemical	Technological	Viab. MO	Sensory
Powders and flours							
[58]	Pineapple	Bark	↑ Antimutagenic Act. ↑ Antioxidant capacity (DPPH and ABTS)	↑ [ ] peptides	↑ AT ↓ pH	↑	-
[59]	Purple grape	Pulp and bagasse	↑ Minerals, dietary fiber	↑ [ ] total phenolic compounds	↑ Viscosity	-	↑ general
[60]	Orange	Albedo	↑ Dietary fiber	-	↓ Syneresis ↑ Protein stability	-	↓ general
[61]	Purple grape	Bagasse	↑ Dietary fiber; ↑ Antioxidant capacity	↑ [ ] total phenolic compounds	↓ Syneresis, ↑ Viscosity	-	↑ general
[62]	Pomegranate and rosewood	Seed	↑ PUFA, conjugated fatty acids ↑ Antioxidant capacity	-	-	↑ Probiotic viability	↑ general
[63]	Apple	Bagasse	-	-	↑ Texture ↑ Stability, ↑ firmness	-	-
[64]	Mango and banana	Bark	↑ Macronutrients ↑ Antioxidant capacity	↑ [ ] total phenolic compounds	-	↑ Probiotics	-
[65]	White mulberry	Pulp	↑ Antioxidant capacity	↑ [ ] total phenolic compounds	-	↑	↑ general
[66]	Sweet potato	Pulp	↑ Antioxidant capacity	↑ [ ] total anthocyanins	↑ Viscosity, ↓ Syneresis	↑	IN
[67]	Pomegranate and grape	Seed	↓ Antioxidant capacity (DPPH and ABTS)	↑ [ ] of total phenolic compounds	↓ Viscosity, ↑ Syneresis	↑	↑ general
[68]	Hemp	Bagasse	↑ Antioxidant capacity	↑ [ ] total phenolic compounds	↑ pH, ↓ Titratable acidity	-	↑
[69]	Date	Pulp	-	-	↑ Hardness ↑ Elasticity ↑ Cohesiveness, ↑ pH, ↓ Acidity ↑ CRA	-	-
[70]	Damascus	Pulp	-	-	↓ Firmness, ↓ pH, ↓ Acidity	↓	-
[71]	Bilberry	Bagasse	↑ Fiber	↑ [ ] total phenolic compounds ↑ [ ] of total anthocyanins	↑ Viscosity, ↓ Syneresis, ↑ CRA	-	↑ general
[72]	Lentinula edodes	Pulp	↑ Antioxidant capacity	-	↓ Viscosity, ↓ Syneresis	↓	-
[73]	Black carrot	Bagasse	↑ Antioxidant capacity	↑ [ ] total phenolic compounds ↑ [ ] total flavonoids ↑ [ ] anthocyanins	↓ Syneresis	-	↑ general
Extracts							
[74]	Pomegranate	Bark	↑ Antioxidant capacity (DPPH and ABTS)	↑ [ ] phenolic compounds and flavonoids	↓ Viscosity ↑ Syneresis	-	↑ general
[75]	*Camellia sinensis*	Sheet	↑ Antioxidant capacity (DPPH and FRAP)	↑ [ ] total phenolic compounds	-	↓	-
[76]	Red beet and lemon	Pulp	↑ Antioxidant capacity	↑ [ ] betalains, carotenoids	-	-	↑ color
[77]	Nutmeg, black pepper, and white pepper	Pulp	↑ Antioxidant capacity	↑ [ ] total phenolic compounds ↑ [ ] peptides	-	-	↑ with black pepper
[78]	Walnuts, pistachios, and almonds	Bark	↑ Antioxidant capacity	-	↓ Syneresis ↑ Stability	↓	↓ for nuts and almonds
[79]	Elderberry	Pulp	↑ Antimicrobial activity ↑ Antioxidant capacity	↑ [ ] total phenolic compounds, flavonoids	-	↑ beneficial, ↓ pathogenic	↑ general
Juices and purees							
[80]	Strawberry, raspberry, and cherry	Pulp	-	↑ [ ] ellagic acid	↑ Rheological properties ↓ pH	↓	↑ color
[81]	Watermelon	Pulp	↑ Proteins, fats, minerals, vitamin C	-	-	-	↑ general
[82]	Passion fruit	Pulp	↑ Antioxidant capacity	↑ [ ] total phenolic compounds	↓ Cohesion	↓	↑ general
[83]	Pomegranate, strawberry, and red beetroot	Pulp	↑ Vitamin C ↑ Antioxidant capacity	↑ total phenolic compounds	↑ Viscosity, ↓ Syneresis ↑ Acidity	↑	↑ general
[84]	Chokeberry	Pulp	↑ Antioxidant capacity	↑ [] total phenolic compounds	↓ pH	-	-
[85]	Strawberries and chia	Pulp and seeds	↑ PUFAS ↑ Fiber	-		-	↑
Emulsions and encapsulates							
[86]	Pinhão	Nanosuspension	↑ Total fibers ↑ Iron ↑ Calcium ↑ Antioxidant capacity (DPPH and ABTS)	↑ [ ] total phenolic compounds	-	-	-
[87]	Cinnamon, garlic, and cumin	Microencapsulated oil	-	-	↑ Viscosity ↑ Lifespan	↓	-

Legend: The abbreviations used in the table refer to different effects observed with the addition of functional ingredients to yogurt formulations. The symbols ↑ and ↓ indicate, respectively, an increase and a decrease in the parameters analyzed. The expression [ ] represents the concentration of certain compounds, such as phenolics, flavonoids, or anthocyanins. The acronym Viab. MO refers to the viability of microorganisms, usually probiotics, present in the yogurt. PUFA indicates the presence of polyunsaturated fatty acids. The DPPH, ABTS, and FRAP methods are used to evaluate the antioxidant capacity of the added ingredients. AT corresponds to titratable acidity, while CRA represents the water retention capacity. The symbol (-) was used to indicate missing or inconclusive data. The abbreviation Sheet refers to the leaves of the plant used as an ingredient, as in the case of Camellia sinensis. Finally, the general term applied in the sensory column refers to the overall acceptability of the enriched product based on attributes such as taste, texture, appearance, and aroma.

## Data Availability

No new data were created or analyzed in this study. Data sharing is not applicable to this article.

## Abbreviations

FAO	Food and Agriculture Organization
pH	Hydrogen potential
Min	Minutes
g	Grams
mL	Milliliters
w	Weight
L	Liter
ROS	Oxygen species
DPPH	2,2-difenil-1-picrilhidrazil
FRAP	Ferric reducing antioxidant power
ABTS	2,2′-azino-bis(3-ethylbenzothiazoline-6-sulfonic acid)
ANVISA	National Health Surveillance Agency
EFSA	European Food Safety Authority

## References

[1] Rațu R.N., Veleșcu I.D., Stoica F., Usturoi A., Arsenoaia V.N., Crivei I.C., Postolache A.N., Lipșa F.D., Filipov F., Florea A.M. (2023). Application of Agri-Food By-Products in the Food Industry. Agriculture.

[2] United Nations (UN) (2015). Transforming Our World: The 2030 Agenda for Sustainable Development.

[3] Sustainability Pathways. https://www.fao.org/nr/sustainability/food-loss-and-waste/en/.

[4] The Circularity Gap Report 2023, Amsterdam: Circle Economy. https://cdn.prod.website-files.com/5e185aa4d27bcf348400ed82/63ecb3ad94e12d3e5599cf54_CGR%202023%20-%20Report.pdf.

[5] Comunian T.A., Silva M.P., Souza C.J.F. (2021). The use of food by-products as a novel for functional foods: Their use as ingredients and for the encapsulation process. Trends Food Sci. Technol..

[6] Cangussu L.B., Fronza P., Cavalcanti W.M. (2020). Pós ricos em fibras de subprodutos de frutas tropicais: Uma revisão bibliográfica sobre seus compostos bioativos. RSD.

[7] Milovanovic B., Tomovic V., Djekic I., Miocinovic J., Solowiej B.G., Lorenzo J.M., Barba F.J., Tomasevic I. (2021). Colour Assessment of Milk and Milk Products Using Computer Vision System and Colorimeter. Int. Dairy J..

[8] Adedokun T.O., Matemu A., Höglinger O., Mlyuka E., Adedeji A. (2022). Evaluation of Functional Attributes and Storage Stability of Novel Juice Blends from Baobab, Pineapple, and Black-Plum Fruits. Heliyon.

[9] Bebek Markovinović A., Brdar D., Putnik P., Bosiljkov T., Durgo K., Huđek Turković A., Brčić Karačonji I., Jurica K., Pavlić B., Granato D. (2024). Strawberry Tree Fruits (*Arbutus unedo* L.): Bioactive Composition, Cellular Antioxidant Activity, and 3D Printing of Functional Foods. Food Chem..

[10] Costa M.P., Monteiro M.L.G., Frasao B.S., Silva V.L.M., Rodrigues B.L., Chiappini C.C.J., Conte-Junior C.A. (2017). Consumer Perception, Health Information, and Instrumental Parameters of Cupuassu (*Theobroma grandiflorum*) Goat Milk Yogurts. J. Dairy Sci..

[11] Ou J. (2021). Incorporation of Polyphenols in Baked Products. Adv. Food Nutr. Res..

[12] De Freitas S.T.F., Benvindo-Souza M., Teodoro L.O., Goulart M.M.P., Pinto T.F.E., Azevedo M.O., Valentim A.M., Pereira P.S., Santos L.R.S., Dyszy F.H. (2020). Aspectos taxonômicos da bioprospecção no Brasil: Tendência científica. Oecologia Aust..

[13] Cubides C., Gutiérrez-Cortés C., Suarez H. (2023). Bioprospecting in Food Production: An Approximation of the Current State in Colombia. Rev. Fac. Nac. Agron..

[14] Ahmad I., Hao M., Li Y., Zhang J., Ding Y., Lyu F. (2022). Fortification of Yogurt with Bioactive Functional Foods and Ingredients and Associated Challenges—A Review. Trends Food Sci. Technol..

[15] Rashwan A.K., Osman A.I., Chen W. (2023). Natural Nutraceuticals for Enhancing Yogurt Properties: A Review. Environ. Chem. Lett..

[16] ONU—United Nations Organization (2022). The Sustainable Development Goals in Brazil. United Nations Brazil. https://brasil.un.org/pt-br/sdgs.

[17] Ferrari R. (2015). Writing Narrative Style Literature Reviews. Med. Writ..

[18] Casarin S.T., Porto A.R., Gabatz R.I.B., Bonow C.A., Ribeiro J.P., Mota M.S. (2020). Types of Literature Review: Considerations of the Editors of the Journal of Nursing and Health. JONAH.

[19] Vescovo D., Manetti C., Ruggieri R., Spizzirri U.G., Aiello F., Martuscelli M., Restuccia D. (2025). The Valorization of Potato Peels as a Functional Ingredient in the Food Industry: A Comprehensive Review. Foods.

[20] Liberati A., Altman D.G., Tetzlaff J., Mulrow C., Gøtzsche P.C., Ioannidis J.P., Moher D. (2009). The PRISMA statement for reporting systematic reviews and metanalyses of studies that evaluate health care interventions: Explanation and elaboration. PLoS Med..

[21] FAO (2013). Pegada de Desperdício de Alimentos: Impactos sobre os Recursos Naturais: Relatório Resumido.

[22] Spizzirri U.G., Espósito L., Caputo P., Martuscelli M., Gagliano M., Clodoveo M.L., De Luca G., Rossi C.O., Savastano M., Scarcelli E. (2024). Farinha de Polpa de Alfarroba como Fonte Inovadora de Moléculas Bioativas para a Preparação de Geleias de Alto Valor Agregado. Heliyon.

[23] PNUMA (2024). Relatório do Índice de Desperdício de Alimentos de 2024. Think Eat Save: Acompanhando o Progresso para Reduzir pela Metade o Desperdício Global de Alimentos.

[24] de Souza J.B., de Oliveira Júnior N.J., Maduro M.R., de Lima O.P. (2024). Impactos Da Indústria 4.0 Na Sustentabilidade No Brasil: Uma revisão bibliográfica. Rev. Gestão Secretariado..

[25] Morrone S., Dimauro C., Gambella F., Cappai M.G. (2022). Industry 4.0 and Precision Livestock Farming (PLF): An up-to-Date Overview across Animal Productions. Sensors.

[26] Alcácer V., Cruz-Machado V. (2019). Scanning the Industry 4.0: A Literature Review on Technologies for Manufacturing Systems. JESTECH.

[27] Liu X., Le Bourvellec C., Yu J., Zhao L., Wang K., Tao Y., Renard C.M.G.C., Hu Z. (2022). Trends and Challenges on Fruit and Vegetable Processing: Insights into Sustainable, Traceable, Precise, Healthy, Intelligent, Personalized and Local Innovative Food Products. Trends Food Sci. Technol..

[28] Verma V.K., Kamble S.S., Ganapatia L., Belhadi A., Gupta S. (2023). 3D Printing for Sustainable Food Supply Chains: Modelling the Implementation Barriers. Int. J. Logist. Res. Appl..

[29] Hassoun A., Prieto M.A., Carpena M., Yamine B., Marvin H.J.P., Pallarés N., Barba F.J., Bangar S.P., Chaudhary V., Ibrahim S. (2022). Exploring the Role of Green and Industry 4.0 Technologies in Achieving Sustainable Development Goals in Food Sectors. Food Res. Int..

[30] Valério G., Costa I., Cardines P. Desenvolvimento de iogurte enriquecido com batata Yacon: Uma proposta de alimento funcional | Revista Terra & Cultura: Cadernos de Ensino e Pesquisa. Unifil.br. http://periodicos.unifil.br/index.php/Revistateste/article/view/2591.

[31] Barbosa A.F., Lopes F.J., Silva V.R.O., Silva M.H.L., Minim V.P.R., Silva R.C.S.N. (2013). Sensory Acceptance of Peach-Flavored Yogurt Supplemented with Different Aroma and Pulp Concentrations Assessed by the Preference Mapping Technique. Rev. ILCT.

[32] Lollo P.C.B., de Moura C.S., Morato P.N., Cruz A.G., Castro W.d.F., Betim C.B., Nisishima L., Faria J.d.A.F., Maróstica M., Fernandes C.O. (2013). Probiotic Yogurt Offers Higher Immune-Protection than Probiotic Whey Beverage. Food Res. Int..

[33] da Silva V.S., Orlandelli R.C. (2019). DESENVOLVIMENTO de ALIMENTOS FUNCIONAIS NOS ÚLTIMOS ANOS: UMA REVISÃO. Rev. Uningá.

[34] Rosa L.d.s., da Cruz A.G., Teodoro A.J. (2022). Produtos Lácteos Probióticos E Câncer—Uma Revisão Narrativa. Res. Soc. Dev..

[35] Plasek B., Lakner Z., Kasza G., Temesi Á. (2019). Consumer Evaluation of the Role of Functional Food Products in Disease Prevention and the Characteristics of Target Groups. Nutrients.

[36] Souto C.N., De Vida E.Q. (2020). Doenças Crônicas: Possíveis Relações / Quality of Life and Chronic Diseases: Possible Relationships. Braz. J. Hea. Rev..

[37] Stover P.J., Garza C., Durga J., Field M.S. (2020). Emerging Concepts in Nutrient Needs. J. Nutr..

[38] Alongi M., Anese M. (2021). Re-Thinking Functional Food Development through a Holistic Approach. J. Funct. Foods.

[39] Mercado L.T.D. https://www.mordorintelligence.com/pt/industry-reports/dairy-products-market.

[40] Guimarães J.T., Balthazar C.F., Silva R., Rocha R.S., Graça J.S., Esmerino E.A., Silva M.C., Sant’Ana A.S., Carmela M., Freitas M.Q. (2019). Impact of Probiotics and Prebiotics on Food Texture. Curr. Opin. Food Sci..

[41] Embrapa Gado de Leite (CNPGL) ANUÁRIO Leite 2022: Pecuária leiteira de precisão. Juiz de Fora: CNPGL, p.114. http://www.infoteca.cnptia.embrapa.br/infoteca/handle/doc/1144110.

[42] Nagaoka S. (2018). Yogurt Production. Methods in Molecular Biology.

[43] Castro D., Teodoro A. (2015). Anticancer Properties of Bioactive Compounds of Berry Fruits—A Review. Br. J. Med. Med. Res..

[44] Cozzolino S.M.F. (2020). Bioavailability of Nutrients.

[45] Rashwan A.K., Karim N., Shishir M.R.I., Bao T., Lu Y., Chen W. (2020). Jujube Fruit: A Potential Nutritious Fruit for the Development of Functional Food Products. J. Funct. Foods.

[46] Rashwan A.K., Karim N., Xu Y., Cui H., Fang J., Cheng K., Mo J., Chen W. (2022). Chemical Composition, Quality Attributes and Antioxidant Activity of Stirred-Type Yogurt Enriched with *Melastoma Dodecandrum* Lour Fruit Powder. Food Funct..

[47] Buchilina A., Aryana K. (2021). Physicochemical and Microbiological Characteristics of Camel Milk Yogurt as Influenced by Monk Fruit Sweetener. J. Dairy Sci..

[48] Huang K., Liu Y., Zhang Y., Cao H., Luo D., Yi C., Guan X. (2022). Formulation of Plant-Based Yoghurt from Soybean and Quinoa and Evaluation of Physicochemical, Rheological, Sensory and Functional Properties. Food Biosci..

[49] Shahein M.R., Atwaa E.S.H., Radwan H.A., Elmeligy A.A., Hafiz A.A., Albrakati A., Elmahallawy E.K. (2022). Production of a Yogurt Drink Enriched with Golden Berry (Physalispubescens L.) Juice and Its Therapeutic Effect on Hepatitis in Rats. Fermentation.

[50] Arroyo B.J., Santos A.P., Almeida de Melo E., Campos A., Lins L., Boyano-Orozco L.C. (2018). Bioactive Compounds and Their Potential Use as Ingredients for Food and Its Application in Food Packaging. Bioactive Compounds: Health Benefits and Potential Applications.

[51] Šeregelj V., Pezo L., Šovljanski O., Lević S., Nedović V., Markov S., Tomić A., Čanadanović-Brunet J., Vulić J., Šaponjac V.T. (2020). New Concept of Fortified Yogurt Formulation with Encapsulated Carrot Waste Extract. LWT.

[52] Saini A., Panwar D., Panesar P.S., Bera M.B. (2020). Encapsulation of Functional Ingredients in Lipidic Nanocarriers and Antimicrobial Applications: A Review. Environ. Chem. Lett..

[53] Silva F.A., do Egypto R.D.C.R., Leite E., Voss G.B., Campelo S., dos Santos Lima M., Manuela M., Vasconcelos S. (2021). Incorporation of Phenolic-Rich Ingredients from Integral Valorization of Isabel Grape Improves the Nutritional, Functional and Sensory Characteristics of Probiotic Goat Milk Yogurt. Food Chem..

[54] Wu T., Deng C., Luo S., Liu C., Hu X. (2023). Effect of Rice Bran on Properties of Yogurt: Comparison between Addition of Bran before Fermentation and after Fermentation. Food Hydrocoll..

[55] Kerdudo A., Burger P., Merck F., Dingas A., Rolland Y., Michel T., Fernandez X. (2016). Development of a Natural Ingredient—Natural Preservative: A Case Study. Comptes Rendus Chim..

[56] Roobab U., Khan A.W., Lorenzo J.M., Arshad R.N., Chen B.-R., Zeng X.-A., Bekhit A.E.-D., Suleman R., Aadil R.M. (2021). A Systematic Review of Clean-Label Alternatives to Synthetic Additives in Raw and Processed Meat with a Special Emphasis on High-Pressure Processing (2018–2021). Food Res. Int..

[57] Baglio E. (2014). The Industry of Yoghurt: Formulations and Food Additives. Chemistry and Technology of Yoghurt Fermentation.

[58] Sah B.N.P., Vasiljevic T., McKechnie S., Donkor O.N. (2015). Effect of Refrigerated Storage on Probiotic Viability and the Production and Stability of Antimutagenic and Antioxidant Peptides in Yogurt Supplemented with Pineapple Peel. J. Dairy Sci..

[59] Karnopp A.R., Oliveira K.G., de Andrade E.F., Postingher B.M., Granato D. (2017). Optimization of an Organic Yogurt Based on Sensorial, Nutritional, and Functional Perspectives. Food Chem..

[60] Kieserling K., Vu T.M., Drusch S., Schalow S. (2019). Impact of Pectin-Rich Orange Fibre on Gel Characteristics and Sensory Properties in Lactic Acid Fermented Yoghurt. Food Hydrocoll..

[61] Demirkol M., Tarakci Z. (2018). Effect of Grape (Vitis Labrusca L.) Pomace Dried by Different Methods on Physicochemical, Microbiological and Bioactive Properties of Yoghurt. LWT.

[62] Van Nieuwenhove C.P., Moyano A., Castro-Gómez P., Fontecha J., Sáez G., Zárate G., Pizarro P.L. (2019). Comparative Study of Pomegranate and Jacaranda Seeds as Functional Components for the Conjugated Linolenic Acid Enrichment of Yogurt. LWT.

[63] Wang X., Kristo E., LaPointe G. (2020). Adding Apple Pomace as a Functional Ingredient in Stirred-Type Yogurt and Yogurt Drinks. Food Hydrocoll..

[64] Zahid H.F., Ranadheera C.S., Fang Z., Ajlouni S. (2022). Functional and Healthy Yogurts Fortified with Probiotics and Fruit Peel Powders. Fermentation.

[65] Sheikh S., Siddique F., Ameer K., Ahmad R.S., Hameed A., Ebad A., Mohamed Ahmed I.A., Shibli S. (2022). Effects of White Mulberry Powder Fortification on Antioxidant Activity, Physicochemical, Microbial and Sensorial Properties of Yogurt Produced from Buffalo Milk. Food Sci. Nutr..

[66] da Cunha Júnior P.C., Pinto C.A.C., Saraiva J.M.A., Ferreira E.H.d.R. (2025). Effects of Purple-Fleshed Sweet Potato Lyophilized Powder on the Physicochemical Properties, Lactic Acid Bacteria Viability, Microstructure, and Textural Properties of Stirred Yogurt. Foods.

[67] Çalişkanlar S., Saygili D., Karagözlü N., Karagözlü C. (2023). Utilization of Pomegranate and Black Grape Seed By-Products in Yogurt Production: Effects on Phenolic Compounds and Antioxidant Activity. Food Sci. Nutr..

[68] Nakov G., Trajkovska B., Atanasova-Pancevska N., Daniloski D., Ivanova N., Lučan Čolić M., Jukić M., Lukinac J. (2023). The Influence of the Addition of Hemp Press Cake Flour on the Properties of Bovine and Ovine Yoghurts. Foods.

[69] Alqahtani N.K., Alnemr T.M., Alsalem A.K., Alotaibi M.M., Mohammed M. (2023). Experimental Investigation and Modeling for the Influence of Adding Date Press Cake on Drinkable Yogurt Quality. Foods.

[70] Karaca O.B., Güzeler N., Tangüler H., Yaşar K., Akın M.B. (2019). Effects of Apricot Fibre on the Physicochemical Characteristics, the Sensory Properties and Bacterial Viability of Nonfat Probiotic Yoghurts. Foods.

[71] Blejan A.M., Nour V., Corbu A.R., Codină G.G. (2024). Influence of Bilberry Pomace Powder Addition on the Physicochemical, Functional, Rheological, and Sensory Properties of Stirred Yogurt. Gels.

[72] Zhu H., Chen Z., Li G., Yao X., Hu Y., Zhao W. (2023). Physicochemical, Sensory, and Antioxidant Characteristics of Stirred-Type Yogurt Enriched with *Lentinula Edodes* Stipe Powder. Food Sci. Nutr..

[73] Stoica F., Rațu R.N., Motrescu I., Cara I.G., Filip M., Țopa D., Jităreanu G. (2024). Application of Pomace Powder of Black Carrot as a Natural Food Ingredient in Yoghurt. Foods.

[74] El-Said M.M., Haggag H.F., El-Din H.M.F., Gad A.S., Farahat A.M. (2014). Antioxidant Activities and Physical Properties of Stirred Yoghurt Fortified with Pomegranate Peel Extracts. Ann. Agric. Sci..

[75] Muniandy P., Shori A.B., Baba A.S. (2016). Influence of Green, White and Black Tea Addition on the Antioxidant Activity of Probiotic Yogurt during Refrigerated Storage. Food Packag. Shelf Life.

[76] dos Santos J., Vasconcelos M.d.F.M., Oliveira G.L.S.d., Silva V.d.C., Júnior B.I.D., Pagani A.A.C. (2020). Avaliação Dos Compostos Bioativos E ação Antioxidante Do Iogurte De Beterraba Com limão/Avaliação dos Compostos Bioativos e Ação Antioxidante do Iogurte de Beterraba com Limão. Braz. J. Desenvolver..

[77] Shori A.B. (2022). Storage Quality and Antioxidant Properties of Yogurt Fortified with Polyphenol Extract from Nutmeg, Black Pepper, and White Pepper. Electron. J. Biotechnol..

[78] Dogan C., Dogan N. (2022). Alterações nas características de qualidade de iogurtes de frutas funcionais fortificados com extratos de casca de várias nozes durante o armazenamento a frio. J. Microb. Biotech. Food. Sci..

[79] Pascariu O.-E., Estevinho L.M., Seixas N.L., Dopcea I., Boiu-Sicuia O.A., Geicu-Cristea M., Israel-Roming F. (2025). Antioxidant Properties and Microbiological Stability of Yogurt Enriched with Elderberry Extract. Foods.

[80] Bueno L., Silva T.M.S., Perina N.P., Bogsan C., Oliveira M.N. (2014). Addition of Strawberry, Raspberry and “Pitanga” Pulps Improves the Physical Properties of Symbiotic Yoghurts. Chem. Eng. Trans..

[81] Reka M., Vijayanchali S.S., Jancy Rani D., Rajapriya K., Nithya R. (2022). Nutrient Composition, Antioxidant Activity and Phytonutrient of Yogurt Incorporated With Watermelon Fruit Pulp and Its Extract. JOAASR.

[82] Ning X., Luo Z., Chen Z., Zhou C., Xie C., Du W., Wang L. (2021). Fortification of Set Yogurt with Passion Fruit Juice: Effects on Fermentation Kinetics, Physicochemical Properties, and Functionality. J. Dairy Sci..

[83] Basiony M., Saleh A., Hassabo R., AL-Fargah A. (2023). The Effect of Using Pomegranate and Strawberry Juices with Red Beet Puree on the Physicochemical, Microbial and Sensory Properties of Yoghurt. J. Food Meas. Charact..

[84] Pădureţ S., Ghinea C., Prisacaru A.E., Leahu A. (2024). Physicochemical, Textural, and Antioxidant Attributes of Yogurts Supplemented with Black Chokeberry: Fruit, Juice, and Pomace. Foods.

[85] Kowaleski J., Quast L.B., Steffens J., Lovato F., Rodrigues dos Santos L., Zambiazi da Silva S., Maschio de Souza D., Felicetti M.A. (2020). Functional Yogurt with Strawberries and Chia Seeds. Food Biosci..

[86] Sbruzzi Fiebig M., Regina Mendes Andrade D., José de Oliveira Mindelo L., Santos de Gois J., Luna A.S., Afonso Provenzi M., Luiz Esteves Magalhães W., Miotto M., Vieira Helm C., Schwinden Prudencio E. (2024). Pinhão Potential and Their Parts (Failures, Shells, and Almonds) in the Elaboration of Yogurts Containing Acai Pulp: Physicochemical, Nutritional, and Functional Properties, Antimicrobial Activity, and Multi-Elemental Profile. Food Res. Int..

[87] Rifky M., Jesfar M., Dissanayake K., Orif U., Samadiy M. (2024). Production of Yoghurts with the Addition of Microencapsulated Cinnamon, Garlic and Cumin Oil with Corn Oil. E3S Web of Conf..

[88] Grimaldi M., Pitirollo O., Ornaghi P., Corradini C., Cavazza A. (2022). Valorization of Agro-Industrial Byproducts: Extraction and Analytical Characterization of Valuable Compounds for Potential Edible Active Packaging Formulation. Food Packag. Shelf Life.

[89] Areia B., Ribeiro J.S., Bruno E., Camelo C., Zanuto M.E. (2022). Principias Métodos de Secagem Utilizados Na Obtenção de Polpa de Fruto Em Pó Solúveis: Uma Revisão. Braz. Appl. Sci. Rev..

[90] Cardoso C.E.F., Lobo F.A.T.F., Teodoro A.J. (2024). Influence of Foam Mat Drying on the Nutritional and Technological Potential of Fruits—A Review. Crit. Rev. Food Sci. Nutr..

[91] de Almeida J.I.O., Costa F., Paulino C.G., de Almeida M.J.O., Damaceno M.N., dos Santos S.M.L., de Farias V.L. (2020). Efeito da Pasteurização nos Compostos Bioativos e na Atividade Antioxidante do Ziziphus Joazeiro Mart Polpa de frutas. RSD.

[92] Yusoff I.M., Taher Z.M., Rahmat Z., Chua L.S. (2022). A Review of Ultrasound-Assisted Extraction for Plant Bioactive Compounds: Phenolics, Flavonoids, Thymols, Saponins and Proteins. Food Res. Int..

[93] Damodaran S., Parkin K.L. (2018). Química de Alimentos de Fennema.

[94] (2000). Resolution No. 5 of November 13, 2000. Standards of Identity and Quality (PIQ) of Fermented Milks, DOU, November 15, 2000. Ministry of Agriculture and Supply. Secretariat of Agricultural Defense. Department of Inspection of Animal Products. Brasilia, Official Gazette of the Union. https://www.dgav.pt/wp-content/uploads/2021/05/Res_5_2000_PIQ_Leites_Fermentados.pdf.

[95] Brückner-Gühmann M., Benthin A., Drusch S. (2018). Enrichment of Yoghurt with Oat Protein Fractions: Structure Formation, Textural Properties and Sensory Evaluation. Food Hydrocoll..

[96] Chen B., Zhao X., Cai Y., Jing X., Zhao M., Zhao Q., Van P. (2022). Incorporation of Modified Okara-Derived Insoluble Soybean Fiber into Set-Type Yogurt: Structural Architecture, Rheological Properties and Moisture Stability. Food Hydrocoll..

[97] Santos R.A.D., de Lima Rodrigues R., de Lima M.B.D., Nascimento E.B.D., de Carvalho A.M.B., de Almeida Gadelha C.A., Gadelha T.S. (2021). Influence of Aqueous Yam Extract and Goat Milk Casein Powder on the Characteristics of Goat Greek-Style Yogurt. Int. J. Gastron. Food Sci..

[98] Brazil. Ministry of Agriculture, Livestock and Supply (2007). Normative Instruction No. 46. Provides for the Adoption of the Technical Regulation on the Identity and Quality of Fermented Milks. https://www.foodchainid.com/product/mapa-no-46-2007/.

[99] Wijesekara A., Weerasingha V., Jayarathna S., Priyashantha H. (2022). Quality Parameters of Natural Phenolics and Its Impact on Physicochemical, Microbiological, and Sensory Quality Attributes of Probiotic Stirred Yogurt during the Storage. Food Chem. X.

[100] Gilbert A., Turgeon S.L. (2021). Studying Stirred Yogurt Microstructure and Its Correlation to Physical Properties: A Review. Food Hydrocoll..

[101] Wong S.-S., Wicklund R., Bridges J., Whaley J., Koh Y.B. (2019). Starch Swelling Behavior and Texture Development in Stirred Yogurt. Food Hydrocoll..

[102] Cheng H. (2010). Volatile Flavor Compounds in Yogurt: A Review. Crit. Rev. Food Sci. Nutr..

[103] Maria H., Cardello F., Cazellatto A., Moskowitz H. (2025). Unlocking Consumer Preferences: Sensory Descriptors Driving Greek Yogurt Acceptance and Innovation. Foods.

[104] Lee H.S., Song M.W., Kim K.-T., Hong W.-S., Paik H.-D. (2021). Antioxidant Effect and Sensory Evaluation of Yogurt Supplemented with Hydroponic Ginseng Root Extract. Foods.

[105] Thakkar S., Anklam E., Xu A., Ulberth F., Li J., Li B., Hugas M., Sarma N., Crerar S., Swift S. (2020). Regulatory Landscape of Dietary Supplements and Herbal Medicines from a Global Perspective. Regul. Toxicol. Pharmacol..

[106] Ministry of Health (Brazil) (2005). National Health Surveillance Agency. Foods with Functional and/or Health Claims, New Foods/Ingredients, Bioactive Substances and Probiotics.

[107] Ministry of Health (Brazil), National Health Surveillance Agency (1999). Resolution No. 18, of April 30, 1999. Approves the Technical Regulation That Establishes the Basic Guidelines for Analysis and Verification of Functional and/or Health Properties Claimed on Food Labels.

